# Designing Sustainable Materials for Energy Applications

**DOI:** 10.1063/4.0000836

**Published:** 2025-10-27

**Authors:** Alicia M Manjon Sanz, Brooke Richtik, Abby Neill, Nabaraj Pokhrel, Mohit Chandra, Michelle R. Dolgos, Valentino Cooper

**Affiliations:** 1 Oak Ridge National Laboratory, Oak Ridge, Tennessee, USA; 2 University of Calgary, Calgary, Alberta, Canada; 3 Johns Hopkins University, Baltimore, Maryland, USA

## Abstract

Piezoelectric materials generate an electric charge in response to mechanical stress and vice versa. Lead Zirconate Titanate, Pb(Zr_1-*x*_Ti*_x_*)O_3_ (PZT) is the current industry standard and the basis for almost all generators, actuators, sensors and related applications critical in today’s technology. Pb(Zr_0.52_Ti_0.48_)O_3_ shows a piezoelectric coefficient (*d*_33_) of 200-600 pC/N. However, because of the impact of lead on the environment and human health, considerable demands have been placed on the scientific community to discover a sustainable lead-free alternative.

Here, we synthesize and investigate the structure-property relationships of environmentally friendly piezoelectric ceramics using neutron powder diffraction data from POWGEN and guided by computational approaches. Such relationships are critical to the design and optimization of piezoelectric devices. Neutrons offer a good contrast between heavy and light atoms compared to X-rays. So, this work is crucial to investigate the octahedral tilts of these materials adopting the perovskite structure and further understand how the structure has an impact on their physical properties. The results of the structure-property relationships of the following lead-free piezoelectric ceramics will be presented:

The system (1-*x*) BaZr_0.2_Ti_0.8_O_3_ -*x* Ba_0.7_Ca_0.3_TiO_3_, which is the first lead-free piezoelectric material with a significantly high enough *d*_33_ ∼ 620 pC/N at *x* = 0.50, that could replace PZT in certain applications. In this study, we re-investigate its phase diagram as a function of temperature. The solid solution between Bi(Ti_3/8_Fe_2/8_Mg_3/8_)O_3_ (BTFM) and PbTiO_3_ (PT). BTFM-*x*PT shows a competitive *d*_33_ of 145 pC/N, and a high Curie temperature (Tc) of 625-650 °C for 0.625BTFM-0.375PT, which is important for high temperature applications.

Lastly, we are performing density functional theory calculations to predict phase stability and ferroelectric performance of materials.

